# The Protein Level of Rev1, a TLS Polymerase in Fission Yeast, Is Strictly Regulated during the Cell Cycle and after DNA Damage

**DOI:** 10.1371/journal.pone.0130000

**Published:** 2015-07-06

**Authors:** Masashi Uchiyama, Junko Terunuma, Fumio Hanaoka

**Affiliations:** Institute for Biomolecular Science, Faculty of Science, Gakushuin University, Toshima-ku, Tokyo, Japan; Institute of Molecular Genetics IMG-CNR, ITALY

## Abstract

Translesion DNA synthesis provides an alternative DNA replication mechanism when template DNA is damaged. In fission yeast, Eso1 (polη), Kpa1/DinB (polκ), Rev1, and Polζ (a complex of Rev3 and Rev7) have been identified as translesion synthesis polymerases. The enzymatic characteristics and protein-protein interactions of these polymerases have been intensively characterized; however, how these proteins are regulated during the cell cycle remains unclear. Therefore, we examined the cell cycle oscillation of translesion polymerases. Interestingly, the protein levels of Rev1 peaked during G^1^ phase and then decreased dramatically at the entry of S phase; this regulation was dependent on the proteasome. Temperature-sensitive proteasome mutants, such as *mts2-U31* and *mts3-U32*, stabilized Rev1 protein when the temperature was shifted to the restrictive condition. In addition, deletion of *pop1* or *pop2*, subunits of SCF ubiquitin ligase complexes, upregulated Rev1 protein levels. Besides these effects during the cell cycle, we also observed upregulation of Rev1 protein upon DNA damage. This upregulation was abolished when *rad3*, a checkpoint protein, was deleted or when the Rev1 promoter was replaced with a constitutive promoter. From these results, we hypothesize that translesion DNA synthesis is strictly controlled through Rev1 protein levels in order to avoid unwanted mutagenesis.

## Introduction

Translesion synthesis (TLS) is a unique DNA damage tolerance pathway in which damaged lesions are replicated using damaged DNA as a template [1]. TLS is triggered by a stalled replicative polymerase at a DNA lesion. The stalled replication fork leads to mono-ubiquitination of proliferating cell nuclear antigen (PCNA), which recruits TLS polymerases [2]. After the loading of TLS polymerases, TLS is carried out through at least two different steps [3, 4]. The first step is insertion of nucleotides opposite to the lesion; this process is mediated mainly by Y family DNA polymerases. The second step is extension of DNA replication past the lesion; this process is mediated mainly by a B family DNA polymerase, polζ [5]. Neither Y family DNA polymerases nor polζ have a proofreading activity [6, 7], and therefore, the mutation rate of TLS remains high.

The enzymatic characteristics and protein-protein interactions among TLS polymerases have been intensively characterized [8, 9]. DNA polymerase η bypasses ultraviolet (UV)-induced cyclobutane pyrimidine dimers accurately [10]. A paralog of this polymerase, DNA polymerase ι, is able to bypass (6–4) photoproducts at the expense of mutagenesis [11]. *Escherichia coli* DinB and human polymerase κ bypasses the *N* ^2^-position of the deoxyguanosine (dG) adduct [12, 13]. Polη, Polι, and Polκ can form complexes with another Y family DNA polymerase, Rev1 [14]. In addition, Rev1 can form a complex with Polζ [15, 16]. These specific protein interactions are thought to ensure appropriate switching of TLS polymerases depending on the type of DNA damage.

Among the TLS polymerases, Rev1 has unique properties [1]. Its polymerase activity is specific to the G template and preferentially inserts deoxycytidine monophosphate (dCMP) [17, 18]. Besides its unique catalytic activity, Rev1 also has important noncatalytic functions. Indeed, Rev1 has a BRCA1 C-terminal (BRCT) domain in its N-terminus and the *rev1-1* mutant, in which the BRCT motif is disrupted, is nonfunctional [19]. BRCT is often found in proteins involved in checkpoint regulation and serves as a phosphorylated protein-binding domain [20]. Mec1, a budding yeast homolog of ataxia telangiectasia and Rad3-related protein (ATR), phosphorylates Rev1 at its N-terminus, thereby increasing the association of Rev1 with chromatin [21, 22]. The BRCT domain has also been shown to mediate the interaction with PCNA [23, 24]. These findings support the hypothesis that the BRCT domain is important for the regulation of TLS. In addition, the Rev1 BRCT motif is functional when substituted for the *DBF4* BRCT motif [25]. However, Rev1 also has additional potential protein-interaction domains. For example, Rev1 is known to associate with POL32, a component of DNA polymerase δ, *in vitro*, through its polymerase-association domain (PAD) [26]. Additionally, mono-ubiquitinated Rev1 is reported to associate with FAAP20, an integral subunit of the multisubunit Fanconi anemia core complex [27]. These interactions indicate that Rev1 serves as an important regulator of TLS.

In fission yeast, Eso1 (polη), Kpa1/DinB, Rev1, and Polζ (a complex of Rev3 and Rev7) have been identified as TLS polymerases [28, 29]. The interaction between Rev1 and Rev7 was confirmed by a two-hybrid system [14]. Kpa1/DinB has been reported to have a functional interaction with the 9-1-1 complex [30]. Although these reports have described the molecular nature of TLS polymerases from fission yeast, no studies have provided an intensive characterization of the regulation of these TLS polymerases in the cell cycle. However, TLS polymerases have been shown to be expressed during the replication of undamaged DNA in budding yeast [31]. Therefore, elucidation of the function of TLS polymerases during normal cell cycle progression is critical.

Here, we addressed this by analyzing changes in the protein levels of TLS polymerases (Rev1 in particular) during cell cycle progression and at sites of DNA damage. We also examined the role of Rev1 as an assembly factor for the interaction between Eso1 and Polζ, a function that was predicted in a previous experiment *in vitro* [32]. Our data suggested that Rev1 protein levels must be strictly regulated to avoid unnecessary activation of mutagenic TLS.

## Materials and Methods

### Strains

Some publically available fission yeast strains were obtained from NBRP (http://yeast.lab.nig.ac.jp/nig/index_en.html). The fission yeast strains used here are listed in S1 Table. Double mutants were created by genetic crosses. All mutant strains and tagged strains were integrants, and the integrated genes resided on the original gene loci and were under the control of their own promoters. The presence of the tags did not alter the growth conditions of the cells.

### Media

For all biochemical analyses, YES medium (yeast extract, 2% glucose, and appropriate supplements) was used. All plates were 2% agar plates containing 3 μg/mL phloxine B prepared with YES medium or Edinburgh minimal medium (EMM, containing appropriate supplements). Appropriate media (either YES medium or EMM) were used for genetic analyses.

### Genetic analysis

All standard genetic analyses were performed as previously described [33, 34]. To characterize the sensitivity of the cells to DNA damaging agents and to examine genetic interactions, we used serial dilution growth assays as previously described [35], with a minor modification (i.e., the fold dilution was changed to five instead of three). For testing drug sensitivity, appropriate concentrations of drugs were added as the plates were prepared.

### Molecular biology techniques

Unless otherwise stated, all molecular biology techniques were performed as previously described [35]. Transformation of yeast strains was performed using the lithium acetate procedure [36]. For cloning purposes, polymerase chain reaction (PCR) was conducted using either pfu-X polymerase (Greiner, Frickenhausen, Germany) or fusion DNA polymerase (Thermo Fisher Scientific, Waltham, MA, USA) to eliminate potential amplification errors.

### Construction of mutant strains

Temperature-sensitive mutants, gene deletion mutants, and *rev1* mutants were generated as previously described [35, 37], with some modifications. The *Rev1* gene fragment, containing 5′ and 3′ noncoding regions (~1 kb), was amplified by PCR and cloned into the pUC18 vector via a *Sma*I restriction site. The *ura4* marker was inserted into the *Sfo*I site of pUC18-*rev1* for nutritional selection. Site-directed mutagenesis was performed using a KOD plus mutagenesis kit (TOYOBO, Osaka, Japan) according to the manufacturer’s instructions. The pfu-X DNA polymerase was used for PCR-based mutagenesis of the *Rev1* gene. The mutagenized pUC18-*rev1 ura4* plasmid was linearized by a restriction digest with *Mlu*I and transformed into the *rev1Δ* strain. The transformants were plated on 5-fluoroorotic acid (5′FOA)-containing plates, and FOA resistant colonies were selected. The mutation was confirmed by mutation site-specific restriction digest, if possible, or was confirmed by sequence analysis.

### Construction of epitope-tagged strains

C terminal epitope tagging was conducted as previously described [35, 38]. For 13myc tagging, we used the pFA631 vector, which was constructed by replacing the *kan^r^* fragment of pFA6a-13myc-kanMX6 with the *his3* gene fragment. For tagging Rev1, twelve 3× flag tandem repeats were inserted into the pFA631 vector. Alternatively, eight or 16 tandem repeats of V5 were inserted into the pFA64 vector for tagging Pop1, Pop2, Eso1, and Rev7. pFA631 or pFA64 tagging constructs were linearized and transformed into appropriate strains. The possible integrants were examined by PCR and immunoblotting with appropriate antibodies.

### *cdc25* synchronized cell culture

To investigate cell cycle-dependent regulation, the epitope-tagged strains were modified as *cdc25* temperature-sensitive mutants. The actual synchronization steps were performed as described previously [35]. *cdc25* temperature-sensitive mutant strains were grown at 25°C, the temperature was shifted to 36°C, and cells were arrested for 2.5 h. Then, the temperature was shifted down to 23°C, and samples of synchronized cells were collected every 20–30 min. The septation index was determined using a microscope. The chromatin localization of Pcn1 peaked at the same time as septation index, generally 100–120 min after release. Therefore, we assumed that the S phase and septation occurred almost simultaneously under our experimental conditions.

### Preparation of whole cell extracts

Harvested cells were washed twice with three volumes of cold water and then washed twice with three volumes of EBL buffer (20 mM Hepes, pH 7.6, 150 mM NaCl, 2 mM MgCl_2_, 0.5 mM EDTA, 0.5 mM EGTA, 12.5 mM 2-mercaptoethanol, 1% Triton X-100, and protease inhibitors). To ensure equal amounts of total protein between different time points or fractions, the total volume of cells was adjusted by measuring the weight of the cell pellet for each sample. The cells were then resuspended with one-quarter volume of EBL. Cell suspensions were frozen drop-wise in liquid nitrogen, and the cells were extracted using a Retsch mill mixer (the liquid nitrogen [LiNi] method). DNaseI was then added to a final concentration of 100 U/mL to the cell suspensions, and samples were centrifuged at 15,000 rpm for 15 min. The supernatants were harvested as whole cell extracts. To examine the amount of protein in each sample, we used the boiling method. The harvested cells were resuspended with an equal volume of EBL, and cell suspensions were boiled for 5 min. Approximately 500 μL acid-washed glass beads (Sigma, St. Louis, MO, USA) was then added, and the cells were extracted for 5 min by vortexing with a Retsch mill mixer. Sodium dodecyl sulfate polyacrylamide gel electrophoresis (SDS-PAGE) sample buffer was added directly to the cell emulsions. After boiling for 3 min, the samples were centrifuged at 15,000 rpm for 15 min, and supernatants were used for SDS-PAGE and western blotting as whole cell extracts.

### Preparation of chromatin-bound and chromatin-unbound fractions

Chromatin-bound fractions were prepared as previously described [35, 39]. Harvested cells were washed twice with three volumes of cold water and twice with three volumes of SB buffer (20 mM Hepes, pH 7.6, 1.2 M sorbitol, 150 mM NaCl, 2 mM MgCl_2_, 0.5 mM EDTA, 0.5 mM EGTA, 12.5 mM 2-mercaptoethanol, and protease inhibitors). To ensure equal amounts of total protein between different time points or fractions, the total volume of cells was adjusted by measuring the weight of the cell pellet for each sample. The cells were then resuspended in the same volume of SB buffer containing Zymolyase-100T (Nakarai, Kyoto, Japan) and spheroplasted by 15-min incubation at 30°C. Cells were washed three times with three volumes of SB buffer and resuspended in EBL buffer. Triton-soluble fractions were extracted by incubation at 4°C for 30 min with gentle mixing followed by centrifugation at 15,000 rpm for 15 min. The pellets were washed four times with three volumes of EBL. Then, the pellets were resuspended in three volumes of EBL. DNaseI was added to a final concentration of 100 U/mL to the cell suspensions, and the cell suspensions were thoroughly lysed by sonication and centrifuged at 15,000 rpm for 15 min. The supernatants were used as chromatin-enriched fractions.

### Analysis of protein expression and interactions

The proteins in the extracts were separated by SDS-PAGE and then transferred onto polyvinylidene difluoride (PVDF) membranes using a semidry blotting apparatus. The membranes were subjected to immunoblotting with appropriate antibodies. Anti-flag M2 (Sigma), anti-myc PL14 (MBL, Nagoya, Japan), anti-V5 V5-10 (Sigma), anti-PCNA PC10 (Abcam plc, Cambridge, UK), anti-Cdc13 6F11/2 (Abcam plc), and anti-Cdc2 PSTAIRE (Santa Cruz Biotechnology, Santa Cruz, CA, USA) were used to detect Flag tag, Myc tag, V5 tag, Pcn1, Cdc13, and Cdc2, respectively. In some experiments, Cdc2 was used as a loading control for whole cell extracts because its expression remains constant throughout the cell cycle. For other experiments, Coomassie Brilliant Blue (CBB) staining of the membrane used for western blotting was presented as a loading control. Immunoprecipitation experiments were performed as previously described, with some modifications [35]. For all processes, EBL buffer was used as washing buffer. For immunoprecipitation of FLAG-tagged and V5-tagged proteins, rabbit anti-FLAG (Sigma) and anti-V5 (MBL) polyclonal antibodies were used, respectively. Approximately 1 μg of the respective antibody was incubated with anti-rabbit IgG-immobilized magnetic beads (Thermo Fisher Scientific). Cell extracts were then incubated with antibody-bound magnetic beads, and the resulting protein-bound magnetic beads were washed at least four times with three volumes of EBL. The supernatant was carefully removed, and the magnetic beads were directly resuspended in 1× SDS-PAGE sample buffer. Then, the immunoprecipitated samples were subjected to SDS-PAGE and western blotting. The proteins were detected with mouse antibodies, if possible, to avoid the high background staining of IgG on the membrane. When we performed immunoprecipitation, an untagged strain was used as a negative control. However, some of the proteins tended to exhibit high background staining when the target-tagged protein was not present in the extract. In these cases, we performed immunoprecipitation on the tagged strain using anti-rabbit normal IgG antibodies (Cell Signaling Technology, Danvers, MA, USA) as a negative control.

## Results

### Rev1 protein levels peaked during the G_1_ phase of the cell cycle

**Fig 1:**
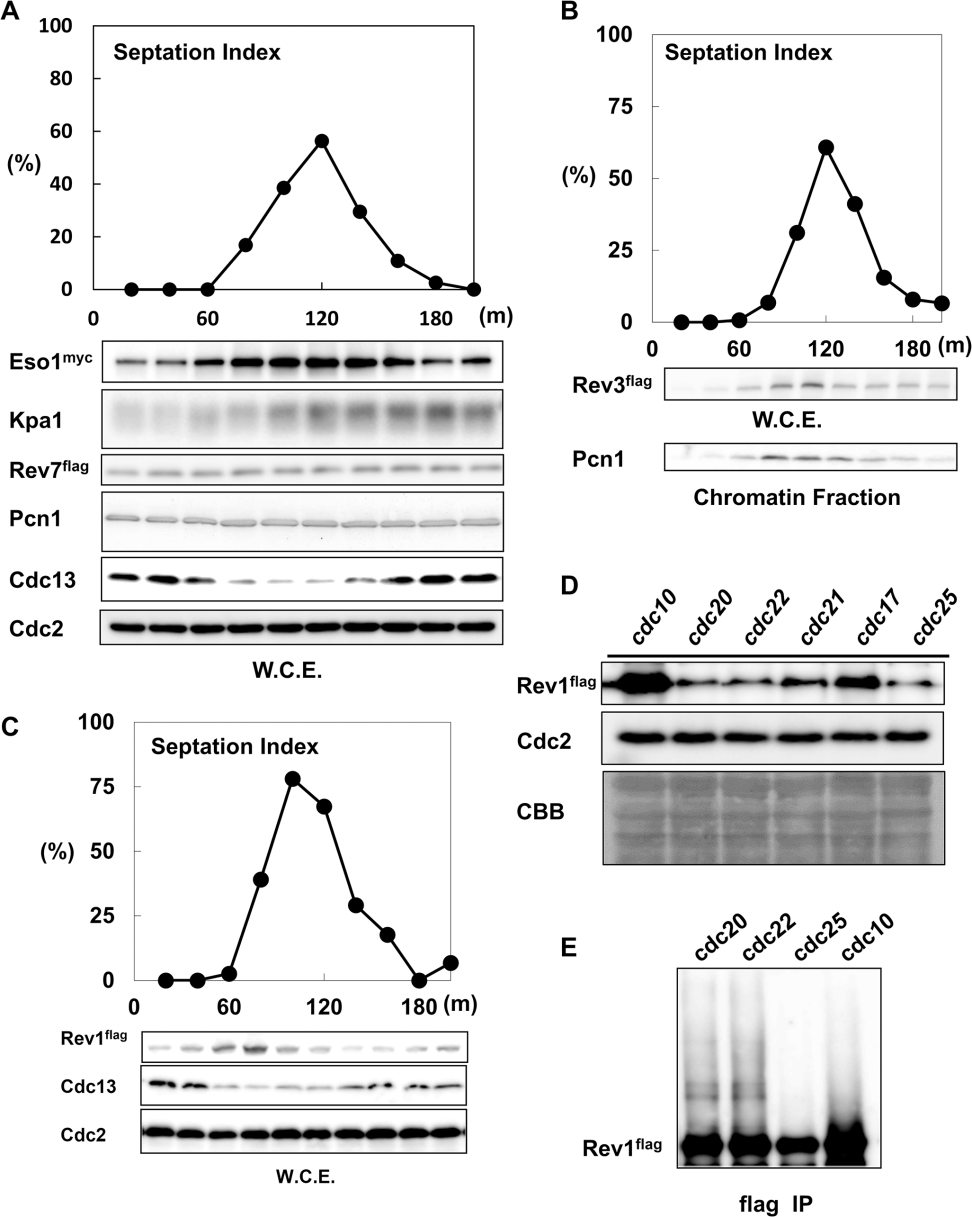
Rev1 protein was most abundant in G^1^ phase. **A,** The TLS polymerases Eso1 and Kpa1 were upregulated in S phase. Eso1, Kpa1, and Rev7 protein expression levels were examined after *cdc25*-dependent block and release. Whole cell extracts were prepared by the boiling method, and western blotting was performed at different times (20–200 min) after the release. The graph represents the septation index after release. The lower panels show the expression patterns of Eso1, Kpa1, Rev7, Pcn1, Cdc13 (cyclin B), and Cdc2. **B,** Rev3 protein expression peaked during S phase. Whole cell extracts were prepared by the boiling method. The graph represents the septation index after release. The middle panel shows the expression pattern of Rev3, which was detected by anti-flag antibodies. The lower panel shows the protein expression of Pcn1 in the chromatin fraction. **C,** The protein amount of Rev1 was highest in G_1_ phase. Whole cell extracts were prepared by the boiling method. The graph represents the septation index after release. The panels show the expression patterns of Rev1, Cdc13, and Cdc2. **D,** The protein expression of Rev1 in cell cycle mutants. *cdc10*, *cdc20*, *cdc22*, *cdc21*, *cdc17*, and *cdc25* mutants harboring flag-tagged *rev1* were arrested at 36.5°C for 4 h. Whole cell extracts were prepared by the boiling method. The amount of Rev1 was analyzed. **E,** Modified forms of the Rev1 protein were observed only in *cdc20* or *cdc22*. *cdc10*, *cdc20*, *cdc22*, and *cdc25* cells harboring flag-tagged *rev1* were arrested at 36.5°C for 4 h. Whole cell extracts were prepared by the LiNi method and then subjected to immunoprecipitation. Rev1 protein expression in each sample was roughly adjusted, and western blotting was performed.

To determine the role of TLS polymerases in regulating the cell cycle, we first analyzed the protein levels of TLS polymerases in *cdc25*-synchronized cultures. As shown in Fig 1A, the septation index reached a maximum at 120 min after the temperature shift. The peak of Eso1 (DNA polymerase η) expression was observed at 120 min, suggesting that Eso1 protein was most abundant during S phase. The peak of Kpa1 (DNA polymerase κ) expression was observed at 140–160 min, indicating that Kpa1 was abundant during S/G_2_ phase. Rev7, an accessory subunit of DNA polymerase ζ, remained constant throughout the cell cycle. Unlike Rev7, Rev3, the catalytic subunit of DNA polymerase ζ, was expressed at its highest level during S phase (Fig 1B).

Next, we analyzed the chromatin loading of Eso1, Kpa1, and Rev7 (S1 Fig). The chromatin loading patterns of Eso1 and Kpa1 were similar to that of Pcn1, fission yeast PCNA, suggesting that Eso1 and Kpa1 loaded onto the chromatin fraction during S phase. Because TLS occurs during DNA replication, these results indicated that TLS polymerases served as an alternative replication system, even during normal cell cycle progression. In contrast to these TLS polymerases, Rev1 was most abundant at 80 min after the temperature shift (Fig 1C). At this time point, the amount of Cdc13/cyclin B was lowest. Since the amount of Cdc13 is the lowest during G_1_ phase, therefore, we assumed that Rev1 was most abundant in G_1_ phase.

We then examined Rev1 protein levels in *cdc10* (MBF transcription factor complex subunit)-, *cdc20* (catalytic subunit of DNA polymerase ε)-, *cdc22* (ribonucleoside reductase large subunit)-, *cdc21* (MCM4)-, *cdc17* (DNA replication ligase)-, and *cdc25* (tyrosine phosphatase)-arrested cultures. As shown in Fig 1D, Rev1 protein levels were highest after *cdc10*-dependent G_1_ arrest [40]. *cdc20* and *cdc22* arrest the cell cycle at the very early stage of S phase [41]; however, the protein level of Rev1 was already significantly decreased by this phase. Among the mutants, Rev1 protein level was the lowest in the *cdc25* mutant, indicating G_2_ arrest. These data also confirmed that Rev1 protein level peaked during G_1_ phase.

### The lysine-rich region of Rev1 was involved in the regulation of Rev1 protein levels

**Fig 2:**
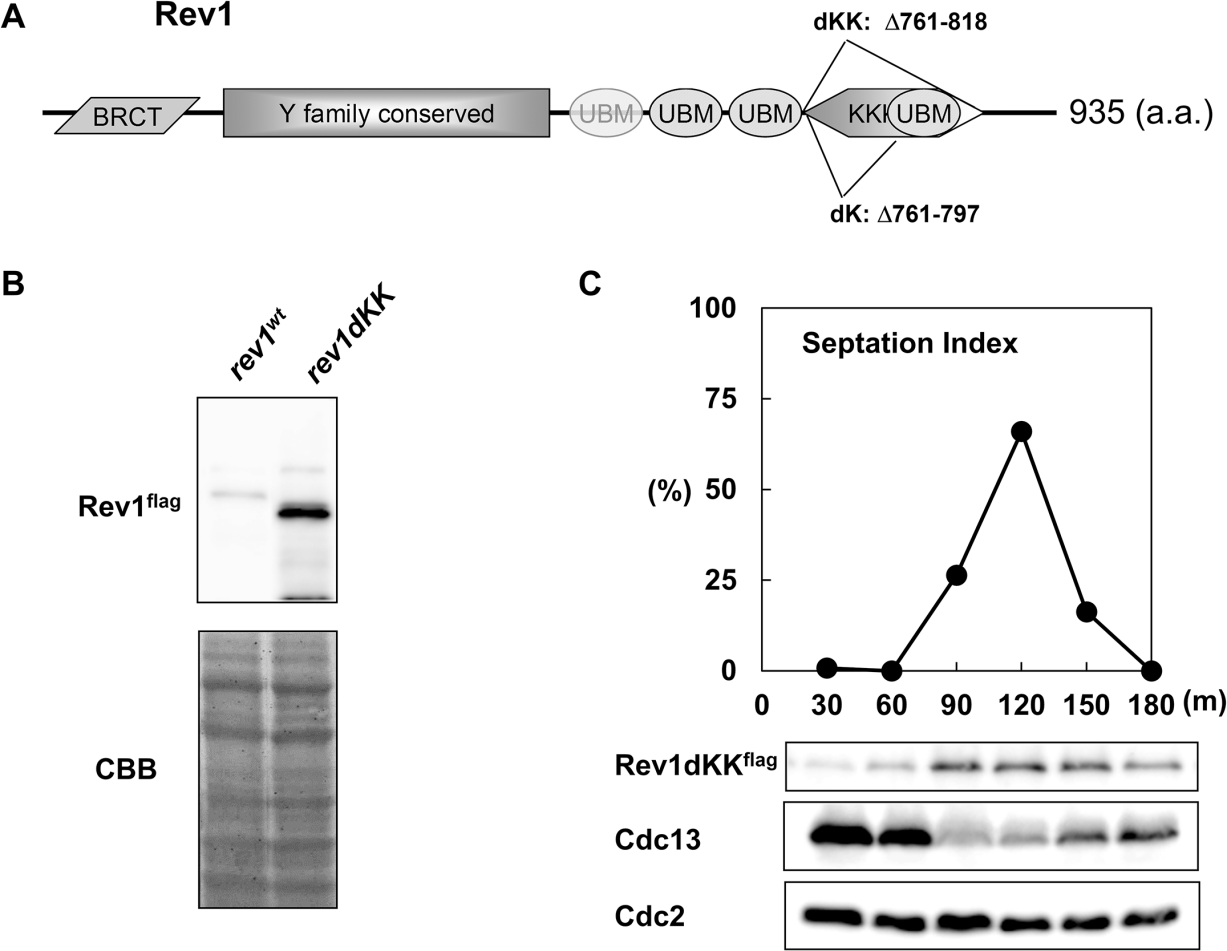
An internal deletion mutant stabilized Rev1 protein in the S phase. **A,** Schematic diagram of the fission yeast Rev1 domain structure. Parallelogram, BRCT; rectangle, Y family-conserved region; ellipse, ubiquitin binding motif; hexagon, Lys-rich region. Rev1dK and Rev1dKK are internal deletion mutants lacking the amino acids from 761 to 818 and 761 to 797, respectively. **B,** The Rev1dKK mutant stabilized Rev1 protein. Flag-tagged *rev1^wt^* and *rev1dKK* cells were grown, and whole cell extracts were prepared by the boiling method. The upper panel shows a western blot of Rev1 protein, and the lower panel shows CBB staining of the membrane as a loading control. The left lane represents Rev1*^wt^*, and the right lane represents Rev1dKK. **C,** Rev1dKK protein was stable in S phase. Time course samples of the *rev1dKK^flag^ cdc25* strain were prepared. The samples were taken every 30 min after the release. The lower panels show the expression patterns of Rev1dKK, Cdc13, and Cdc2.

We found that Rev1 protein levels dropped sharply during G_1_/S phase. Interestingly, a similar destabilization of Rev1 at G_1_/S was observed in budding yeast, and this destabilization was found to be dependent on the proteasome [42]. Therefore, the proteasome may also control Rev1 protein levels in fission yeast. Analysis of Rev1 protein levels (Fig 1C) demonstrated that the band representing Rev1 protein during G_1_/S was often present as a high-molecular-weight smear on western blotting, also supporting the potential proteasome-dependent regulation of Rev1. To examine the nature of this high-molecular-weight band, we immunoprecipitated Rev1 in *cdc10*-, *cdc20*-, *cdc22*-, and *cdc25*-arrested cultures. When the total amount of Rev1 was roughly equalized, the high-molecular-weight bands (resembling a ladder) were observed only in *cdc20*- and *cdc22*-arrested extracts (Fig 1E), suggesting that Rev1 underwent ubiquitin-dependent proteolysis. Ubiquitination targets the lysine residues of the protein; thus, we first constructed Rev1dKK, an internal deletion mutant (Δ761–818) that lacked the lysine-rich region near the C terminus (Fig 2A). As expected, Rev1dKK protein levels were much higher than those of Rev1^wt^, even in unsynchronized conditions (Fig 2B). Next, we examined the protein level of Rev1 in the Rev1dKK mutant in *cdc25*-synchronized cultures. As shown in Fig 2C, Rev1dKK protein levels peaked at S phase instead of G_1_ phase. Because cell cycle-dependent oscillation of *rev1* mRNA was previously reported [43], the observed protein level oscillation of Rev1dKK probably resulted from regulation of the mRNA level. We also created a Rev1dK internal deletion mutant (Δ761–797) because the Rev1dKK mutant lacked a ubiquitin-binding motif, which is important for the function of Rev1. Rev1 protein was also elevated in the Rev1dK mutant compared to the Rev1^wt^ strain (S2 Fig). These data suggested that Rev1 underwent ubiquitin-dependent proteolysis at G_1_/S phase.

### 26S proteasome subunit temperature-sensitive mutants stabilized Rev1 protein

**Fig 3:**
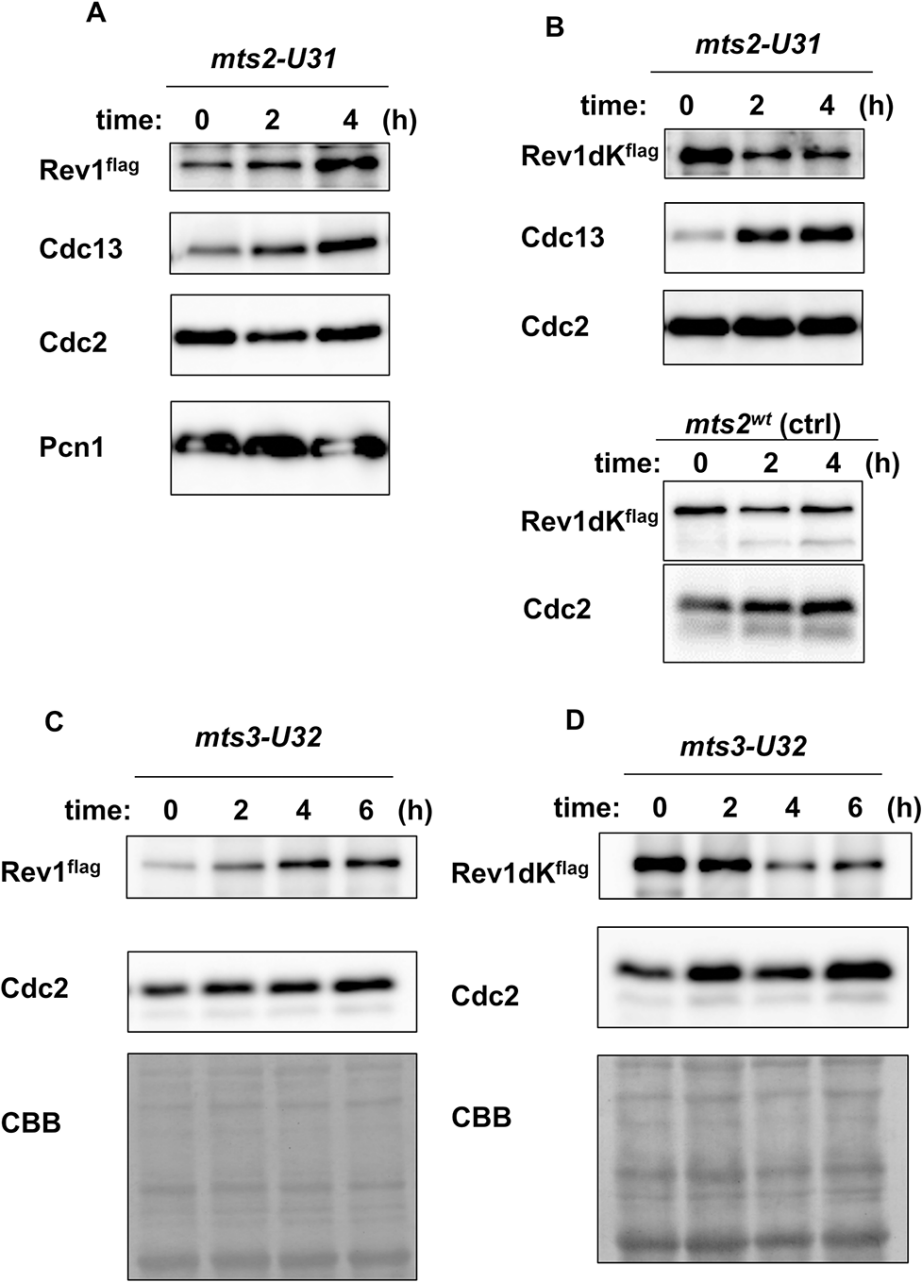
Rev1 protein was stabilized under the restrictive condition for the *mts2* or *mts3* temperature-sensitive mutant. **A,** Protein level of Rev1 was increased after the temperature shift in the *mts2* temperature-sensitive mutant. The *mts2-U31* mutant, harboring-flag tagged Rev1, was first grown at 25°C, and the temperature was then shifted to 36.5°C. Samples were collected every 2 h until 4 h after the shift. Whole cell extracts were prepared. The panels show the protein expression of Rev1, Cdc13, Cdc2, and Pcn1. **B,** The protein level of Rev1dK did not increase at the restrictive temperature of *mts2-U31*. The panels show the protein expression of Rev1dK, Cdc13, and Cdc2 in the *mts2-U31* strain, and that of Rev1dK and Cdc2 in the *mts2^wt^* control. The lanes represent the protein expression at 0, 2, and 4 h after the temperature shift in the *mts2-U31 or mts2^wt^* strain. **C,** The protein level of Rev1 increased after the temperature shift in the *mts3* temperature-sensitive mutant. The *mts3-U32* mutant, harboring flag-tagged Rev1, was first grown at 25°C, and the temperature was then shifted to 36.5°C. The samples were collected every 2 h until 6 h after the shift. Whole cell extracts were prepared. The panels show the protein expression of Rev1 and Cdc2, as well as CBB staining of the membrane. **D,** The protein level of Rev1dK did not increase at the restrictive temperature in the *mts3-U32* strain. The panels show the protein expression of Rev1dK and Cdc2, as well as CBB staining of the membrane. The lanes represent the protein expression at 0, 2, 4, and 6 h after the temperature shift in the *mts3-U32* strain.

Ubiquitin-dependent proteolysis occurs through the activity of proteasome complexes [44, 45]. Thus, we next analyzed Rev1 protein levels in proteasome-deficient conditions. First, we used random mutagenesis to construct the *mts2/rpt2* temperature-sensitive mutant, which disrupted the function of a proteasomal subunit [46] following appropriate changes in temperature. The protein level of Rev1 was then examined at the restrictive condition of the temperature-sensitive mutant. The protein levels of Cdc13, which is controlled by proteasome-dependent proteolysis [47], and Rev1 were increased after *mts2-U31* mutant cells were arrested (Fig 3A). In contrast, the protein levels of the Rev1dK mutant did not increase after *mts2-U31*-dependent cell cycle arrest (Fig 3B). The apparent decrease in the protein level of Rev1dK resulted from the increasing temperature and cell cycle arrest in M phase caused by *mts2-U31*. To further confirm the contribution of the proteasome to Rev1 protein levels, we also constructed *mts3/rpn12* [48] temperature-sensitive mutants. Similarly, Rev1 protein levels increased in *mts3-U32* cells at the restrictive temperature (Fig 3C), and the amount of Rev1dK did not increase under the same conditions (Fig 3D). These data clearly indicated that Rev1 protein levels were controlled by proteasomal degradation.

### Pop1 and Pop2, subunits of the SCF ubiquitin ligase, were responsible for the destruction of Rev1

**Fig 4:**
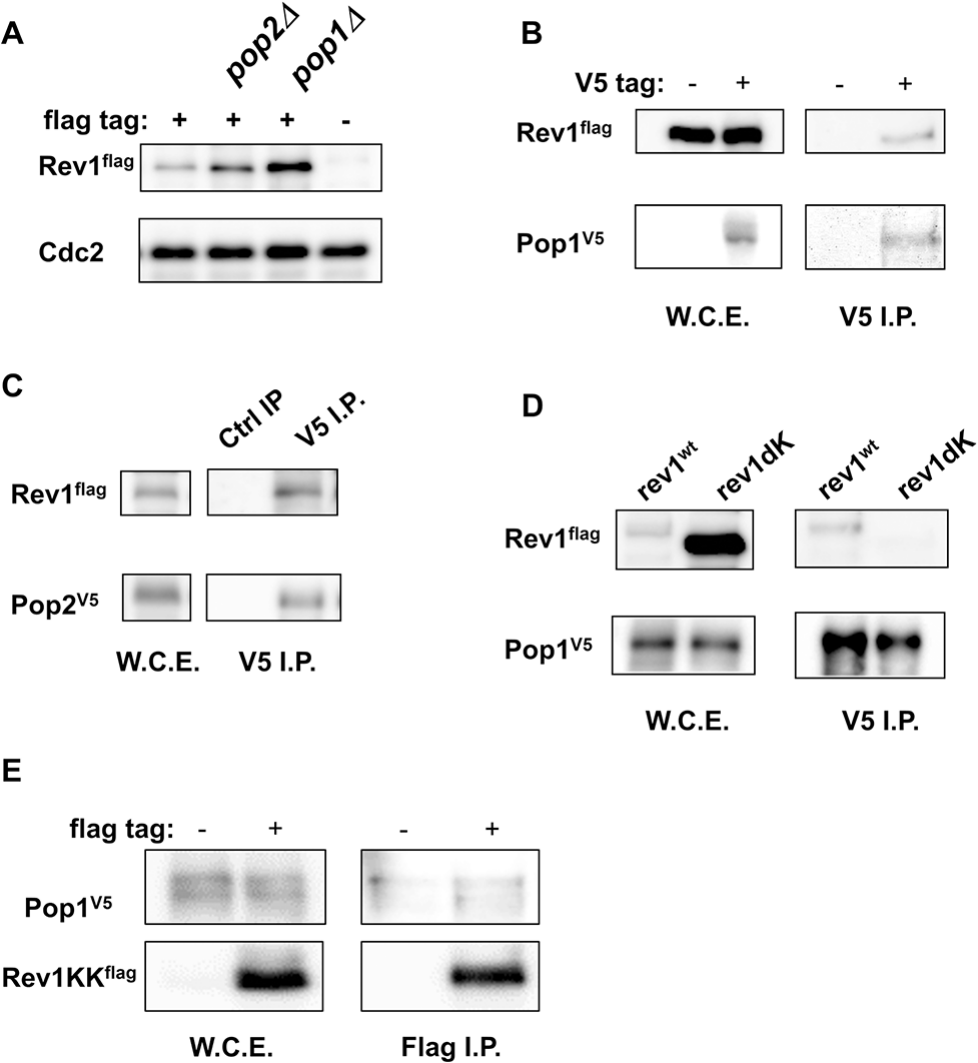
Pop1 and Pop2 were responsible for the stability of Rev1. **A,** *pop1* and *pop2* deletion mutants stabilized Rev1 protein. wt, *pop1Δ*, and *pop2Δ* strains harboring flag-tagged *rev1* and an untagged *rev1* wt strain were grown, and whole cell extracts were prepared by the boiling method. Protein expression levels were compared by western blotting. The upper panel shows the amount of Rev1 in *rev1^flag^*, *rev1^flag^ pop2Δ*, *rev1^flag^ pop1Δ* and untagged *rev1* strains. The lower panel shows Cdc2 as a loading control. **B,** Rev1 was coprecipitated with Pop1. *rev1^flag^ pop1^V5^* and *rev1^flag^* strains were grown, and whole cell extracts were prepared by the LiNi method. Immunoprecipitation was performed using anti-V5 antibodies. The left panels show Rev1 and Pop1 input. The right panels show Rev1 and Pop1 in anti-V5-immunoprecipitated fractions. **C,** Rev1 was co-precipitated with Pop2. The *rev1^flag^ pop2^V5^* strain was grown, and whole cell extracts were prepared. Immunoprecipitation was performed using rabbit normal IgG or anti-V5 antibodies. The left panels show Rev1 and Pop2 input. The right panels show Rev1 and Pop2 in rabbit normal IgG- or anti-V5-immunoprecipitated fractions. **D,** The Rev1dK mutant was not coprecipitated with Pop1. *rev1^flag^ pop1^V5^* and *rev1dK^flag^ pop1^V5^* strains were grown, and whole cell extracts were prepared. Immunoprecipitation was performed using anti-V5 antibodies. The left panels show Rev1 and Pop1 input. The right panel shows Rev1 and Pop1 in anti-V5-immunoprecipitated fractions. **E,** Rev1KK (Rev1 761–818) associated with Pop1. Amino acids 761–818 of Rev1, which is the region deleted in Rev1dKK, were cloned into a pCAM1-flag expression vector. The *pop1^V5^* strain with the pCAM1-*rev1KKflag* expression vector was grown at 30°C and whole cell extracts were prepared. Immunoprecipitation was performed using rabbit normal IgG or anti-V5 antibodies. The left panels show Rev1 and Pop1 input. The right panels show Rev1KK and Pop1 in rabbit normal IgG- or anti-V5-immunoprecipitated fractions.

Next, we examined which ubiquitin ligase may be responsible for the degradation of Rev1. As shown in Fig 1C and 1D, Rev1 protein levels decreased dramatically at the onset of DNA replication. This pattern was similar to that of Cdc18, a functional homolog of budding yeast Cdc6 [49, 50]. The degradation of Cdc18 at G_1_/S depends on Pop1 and Pop2, subunits of SCF ubiquitin ligase [50, 51]. Therefore, we examined the involvement of Pop1 and Pop2 in the degradation of Rev1 at G_1_/S. Since *pop1* and *pop2* genes are not essential, we analyzed the protein levels of Rev1 in the *pop1* or *pop2* deletion background. The protein amount of Rev1 increased both in *pop1Δ* and *pop2Δ* mutants, although the contribution of *pop1Δ* was predominant (Fig 4A). Next, we examined the physical interactions between Rev1 and Pop1 or Pop2. As shown in Fig 4B and 4C, Rev1 was coprecipitated with Pop1 and Pop2. The interaction between Rev1 and Pop1 was also examined in *cdc25*-synchronized culture (S3 Fig). Rev1 was coprecipitated with Pop1, and the interaction peaked at about 100 min after the release. These data supported our hypothesis that Rev1 destruction at G_1_/S was controlled by SCF-dependent ubiquitination. To examine the contribution of the Lys-rich region of Rev1 for SCF-dependent proteolysis, we examined the protein-protein interactions between Pop1 and Rev1dK (Fig 4D). Interestingly, Rev1dK failed to interact with Pop1 following immunoprecipitation of Pop1. This result suggested that Rev1dK might become stable because it cannot interact with ubiquitin ligase. To test this hypothesis, we examined the interactions between Pop1 and Rev1KK (amino acids 761–818 of Rev1) (Fig 4E). Rev1KK was associated with Pop1. Based on these findings, we concluded that Rev1 destruction by the proteasome was mediated by Pop1 or Pop2 SCF ubiquitin ligase.

### Rev1 served as an assembly factor for TLS polymerases

**Fig 5:**
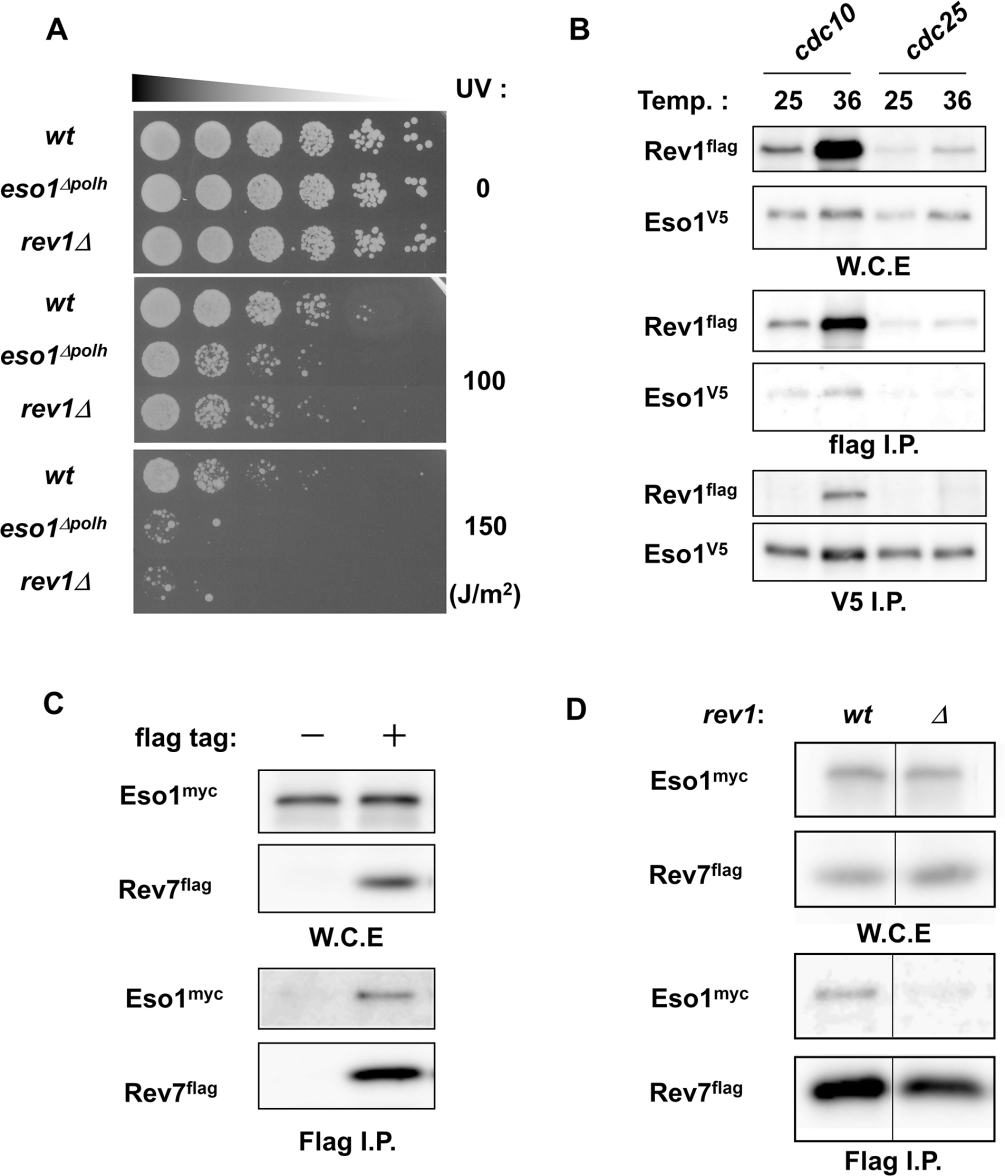
Eso1 and Rev1 were associated in G_1_ phase. **A,** *eso1^Δpolh^* and *rev1Δ* mutants exhibited similar sensitivities to UV irradiation. Cells in the logarithmic growth phase were serially diluted by 5 fold. Cells were then spotted on YES plates and exposed to UV light (0, 100, or 150 J/m*^2^*). Plates were incubated at 30°C, and the growth of wt, *eso1^Δpolh^*, and *rev1Δ* strains was observed 3 days after the irradiation. **B,** Eso1 associated with Rev1 in *cdc10*-arrested extracts. *cdc10 rev1^flag^ eso1^V5^* and *cdc25 rev1^flag^ eso1^V5^* strains were first grown at 25°C, and the cultures were split into two. The first half was further grown at 25°C, and the second half was grown at 36°C for 3 h. The cells were harvested, and whole cell extracts were prepared. Immunoprecipitation was then carried out using anti-flag or anti-V5 antibodies. The panels represent input, Flag IP, and V5 IP of Rev1 and Eso1 protein in the *cdc10* strain at 25°C, the *cdc10* strain at 36.5°C, the *cdc25* strain at 25°C, and the *cdc25* strain at 36.5°C. **C,** Eso1 was coprecipitated with Rev7. *eso1^myc^* and *eso1^myc^ rev7^flag^* strains were grown, and whole cell extracts were prepared. Immunoprecipitation was performed using anti-flag antibodies. The panels show the expression of Eso1 and Rev7 in input samples and immunoprecipitated fractions. **D,** Rev7 failed to coprecipitate Eso1 in the absence of Rev1. *eso1^myc^ rev7^flag^* and *eso1^myc^ rev7^flag^ rev1Δ* strains were grown, and whole cell extracts were prepared. Immunoprecipitation was performed using anti-flag antibodies. The panels show the expression of Eso1 and Rev7 in input samples and immunoprecipitated fractions.

Studies investigating the function of Rev1 in humans and budding yeast have indicated that Rev1 may serve as a scaffold for TLS polymerases [32, 52–54]. The increase in Rev1 protein levels during G_1_ phase may be a prerequisite for the formation of the TLS polymerase complex in S phase, similar to the role of Cdc18 in replicative complexes. To assess this possibility, we examined the relationship between Rev1 and Eso1, a polymerase η homolog. First, we examined the UV sensitivity of strains deficient in Rev1 or polymerase η. The *rev1Δ* mutant conferred cells with a sensitivity similar to that of *eso1^Δpolη^* when cells were irradiated at 100 or 150 J/m^2^ (Fig 5A). The enzymatic function of Rev1 after UV irradiation is not thought to be critical since Rev1 preferentially functions as a dCMP transferase. Therefore, this result suggested that Rev1 served as a regulator of Eso1. Next, we examined the physical interactions between Rev1 and Eso1. When we immunoprecipitated Rev1 or Eso1 from extracts of *cdc10*-arrested cells, Eso1 was co-immunoprecipitated with Rev1, and Rev1 was co-immunoprecipitated with Eso1 (Fig 5B). Therefore, these data confirmed the physical association between Rev1 and Eso1 in G_1_ phase. Next, we examined whether Rev1, polymerase η, and polymerase ζ formed a complex. As reported in other species [55], Rev1 was associated with the polζ subunit Rev7 (S2 Fig). Next, we examined the association of Eso1 with Rev7 in the *rev1* deletion background. As shown in Fig 5C, immunoprecipitation analysis showed that Eso1 associated with Rev7. However, we were unable to detect this association by immunoprecipitation in the *rev1Δ* background (Fig 5D). From these results, we concluded that the fission yeast Rev1 also functioned as a polζ assembly factor for Eso1/polη.

### Excess Rev1 may have negative effects on TLS

**Fig 6:**
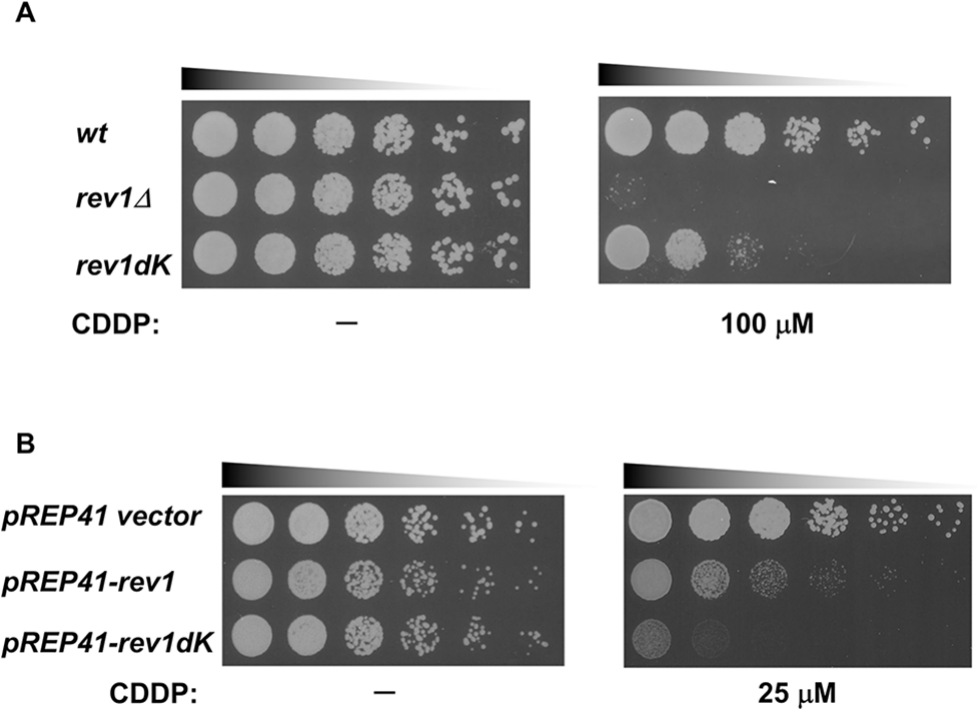
Increased expression of Rev1 sensitized the cells to cisplatin. **A,** *rev1dK* exhibited cisplatin (CDDP) sensitivity. wt, *rev1Δ*, and *rev1dK* strains were grown. Cells were spotted on YES plates containing cisplatin with 5-fold serial dilutions, and the plates were incubated at 30°C for 3 days. The panels represent the growth of wt, *rev1Δ*, and *rev1dK* strains on cisplatin minus control and on a YES plate containing 100 μM cisplatin. **B,** Overexpression of *rev1* conferred sensitivity to cisplatin. Cells harboring pREP41, pREP41-*rev1*, or pREP41-*rev1dK* plasmids were grown in EMM media without thiamine and spotted on an EMM plate containing 0 or 25μM CDDP with 5-fold serial dilutions. The plates were incubated at 30°C for 3 days. The panels represent the growth on an EMM plate without CDDP or containing 25 μM CDDP.

As shown in the previous section, Rev1 may serve as a regulator of TLS. However, it is unclear why Rev1 protein, which was abundantly expressed in G_1_ phase, must be destroyed when cells progress into the S phase. Indeed, Rev1 destruction may have positive effects on TLS as overproduction of the C-term domain of Rev1 has been reported to sensitize budding yeast to DNA damage [56]. To examine this possibility, we decided to analyze the DNA damage sensitivity of *rev1dK*, a proteasome-insensitive mutant. The *rev1* deletion mutant exhibited sensitivities to various DNA-damaging agents (S7 Fig). Among the agents we tested, *rev1Δ* exhibited the highest sensitivity to cisplatin (CDDP); therefore, we utilized cisplatin as a damaging agent. Although weaker than that of *rev1Δ*, *rev1dK* also exhibited cisplatin sensitivity (Fig 6A). Interestingly, as shown in S2, S4, and S5 Figs, the *rev1dK* structure maintained all known functional domains of Rev1 intact; thus, it is highly possible that the cisplatin sensitivity of the *rev1dK* mutant was conferred by the increased amount of Rev1. To investigate this possibility, we examined the effects of Rev1 protein levels on cisplatin sensitivity by overexpressing Rev1. *rev1^wt^* and *rev1dK* were overexpressed under the thiamine-repressive *nmt41* promoter under activation conditions. To confirm the overproduction of Rev1, we also overexpressed a flag-tagged version of *rev1* and the expression level was examined (S6 Fig). Interestingly, both *rev1dK* and *rev1^wt^* overproduction enhanced the cellular sensitivity to cisplatin (Fig 6B), indicating that Rev1 was able to serve as a negative regulator for TLS.

### The checkpoint protein Rad3 was required to control the expression of Rev1 protein after DNA damage

**Fig 7:**
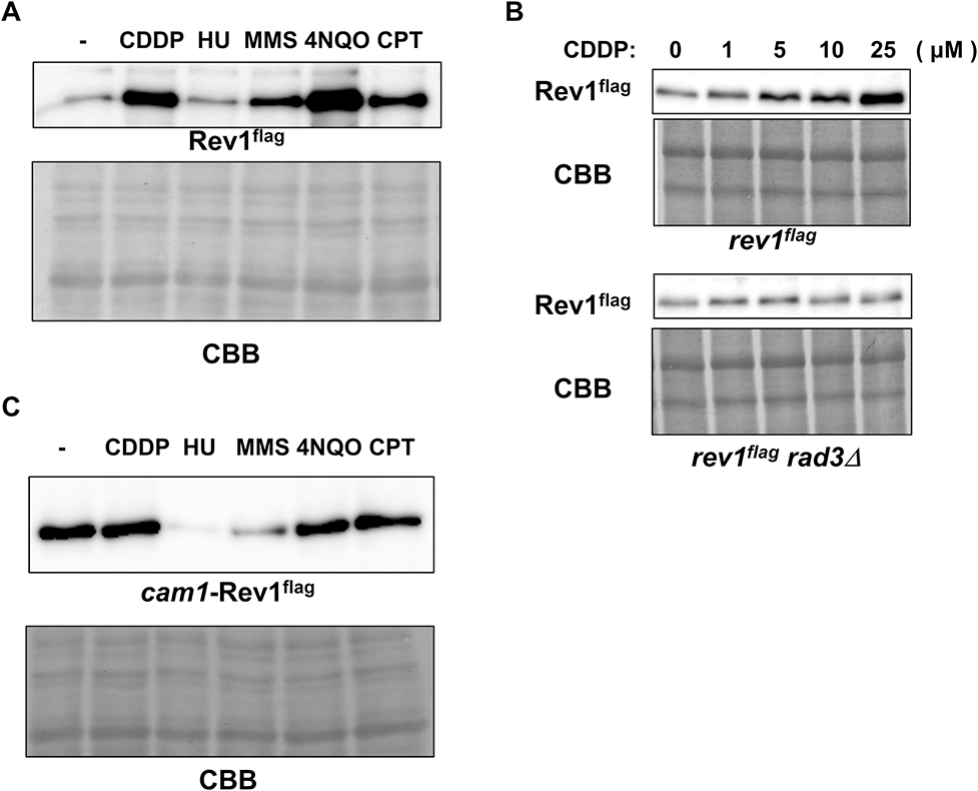
The protein expression of Rev1 was upregulated in response to DNA damage. **A,** Rev1 protein expression after mutagen treatment. At the logarithmic growth phase, wt cells harboring flag-tagged *rev1* were treated with no drug (-), 50 μM cisplatin (CDDP), 10 mM hydroxyurea (HU), 0.008% MMS, 500 nM 4NQO, or 40 μM camptothecin (CPT). After a 4-h incubation, cells were harvested. Whole cell extracts were prepared by the boiling method, and protein levels were examined by western blotting. The upper panel represents flag-tagged Rev1, and the lower panel shows CBB staining of the membrane. **B,** The upregulation of Rev1 was dependent on Rad3. At the logarithmic growth phase, *rev1^flag^* and *rev1^flag^ rad3Δ* strains were treated with 0, 1, 5, 10, or 25 μM cisplatin for 3 h. Cells were then harvested, and whole cell extracts were prepared by the boiling method. Extracts were then subjected to western blotting. The upper panels show Rev1 and CBB staining of the *rev1^flag^* strain, and the lower panels show those of the *rev1^flag^ rad3Δ* strain. **C,** The promoter region was important for the upregulation of Rev1 after DNA damage. At the logarithmic growth phase, *cam1-rev1* cells were treated with no drug (-), 50 μM cisplatin (CDDP), 10 mM hydroxyurea (HU), 0.008% MMS, 500 nM 4NQO, or 40 μM camptothecin (CPT). After a 4-h incubation, cells were harvested, and whole cell extracts were prepared by the boiling method. Protein expression was then examined by western blotting. The upper panel shows Rev1, and the lower panel shows CBB staining.

Because we confirmed the proteasomal regulation of Rev1 and its significance, we next focused on the unexpected upregulation of Rev1 in *cdc21*- and *cdc17*-arrested cultures. Both genes are important for DNA replication. It is possible that DNA damage structures are created because of defects in these gene products. Therefore, we examined whether Rev1 protein expression was altered following induction of DNA damage. As we described above, Rev1 exhibited sensitivity to cisplatin (CDDP), MMS, and 4NQO (S7 Fig). However, Rev1 did not exhibit sensitivity to camptothecin (CPT; S7 Fig). Therefore, we used these DNA-damaging agents and hydroxyurea, which blocks DNA replication, to examine changes in Rev1 expression. As shown in Fig 7A, Rev1 protein was upregulated in all samples treated with DNA-damaging agents, but was not detected in the hydroxyurea-treated sample.

Because checkpoint-dependent upregulation of various proteins has been observed after induction of DNA damage [57], Rev1 protein may be controlled via these same mechanisms. Therefore, we examined Rev1 protein expression after DNA damage in the *rad3Δ* background because *rad3* is essential for checkpoint activation [58]. As shown in Fig 7B, the amount of Rev1 increased depending on the concentration of cisplatin. On the other hand, the expression of Rev1 remained unchanged in the *rad3Δ* background, regardless of the presence of cisplatin. This result clearly indicated that Rev1 expression was upregulated upon DNA damage in a checkpoint-dependent manner.

Cell cycle checkpoints regulate protein expression via several mechanisms, including transcriptional regulation [59]. Therefore, we examined whether this pathway may control Rev1 expression. The *rev1* gene, controlled by the *cam1* promoter, an ectopic constitutively active promoter [60], was integrated into the *rev1Δ* strain. The integrant *cam1-rev1* was treated with DNA-damaging agents, and the protein amount was examined. No increase in Rev1 protein expression was observed in the *cam1-rev1* strain (Fig 7C). This result supported the hypothesis that checkpoint-dependent Rev1 upregulation occurred through transcriptional regulation.

## Discussion

In this study, we examined the oscillation of TLS polymerases in the cell cycle. We found that levels of TLS polymerases were regulated during the cell cycle, even in the absence of DNA-damaging agents. In addition, Eso1 and Kpa1 were abundant in the chromatin fraction during S phase. These data imply that TLS polymerases were integrated into DNA replication systems, even when DNA was not damaged, and served as alternative replication pathways in case the replication machinery encountered regions of damaged DNA. This possibility has been reported previously [31]. However, our data also demonstrated that Rev1, an important TLS polymerase, was downregulated during S phase. We suspected that this unexpected downregulation may be the key to understanding how TLS polymerases serve as alternative replication machinery. Thus, our data identify Rev1 as an important player in TLS and normal cell cycle progression.

In budding yeast, *REV1* deletion mutants have been shown to exhibit sensitivity to UV irradiation [19]. We also found this to be true in fission yeast. *REV1* deletion mutants have also been shown to confer sensitivity to various DNA damage-inducing agents [61, 62]. If we consider these findings, it is clear that *rev1* has an important function in TLS. However, our finding that Rev1 protein levels decreased sharply at the G_1_/S boundary appeared to contradict the current understanding of the function of Rev1. A previous report from Walker’s group showed that Rev1 protein levels are remain very low during S phase compared to that in G_2_/M phase in budding yeast [63]. These findings indicate that downregulation of Rev1 in S phase is a universal mechanism. TLS is thought to function as an alternative DNA replication mechanism in S phase. Therefore, these observations suggest that the contribution of Rev1 to TLS may be minor. However, we also found that the *rev1* deletion mutant exhibited UV sensitivity similar to that of *eso1^Δpolh^*, a DNA polymerase η domain deletion mutant. Therefore, while Rev1 must have an important function in S phase, its expression in S phase must be kept low. This pattern is similar to that of Cdc18, a functional homolog of budding yeast Cdc6 [64, 65]. Cdc18 undergoes SCF-dependent proteolysis at the G_1_/S boundary, but has an essential function in the initiation of DNA replication. Cdc18 interacts with the ORC complex during the M/G_1_ phase [66] and recruits MCM to the initiation sites of chromatin [67]. Subsequently, Cdc18 is destroyed in an SCF-dependent manner after the recruitment of MCM. Thus, these findings suggest that Rev1 is controlled through similar mechanisms. Accordingly, we found that the SCF components Pop1 and Pop2 are responsible for Rev1 destruction at G_1_/S. Moreover, because Cdc18 serves as a loading factor for MCM, Rev1 may also serve as a loading factor for TLS polymerases. Consistent with this notion, we found that Rev1 served as an assembly factor for Eso1 to interact with DNA polymerase ζ. Given that the protein levels of Rev1 increased before the onset of S phase and that other TLS polymerases are upregulated during S phase, it is plausible that chromatin-loaded Rev1 serves as a center for the assembly of TLS polymerases, i.e., in a manner analogous to the mechanism through which Cdc18 acts as a loading factor for MCM.

Here, we identified that the protein level of Rev1 is controlled by SCF and that this regulation is similar to that for Cdc18. The destruction of Cdc18 is triggered by CDK-dependent phosphorylation [49], but it remains unclear whether the destruction of Rev1 is also triggered by CDK-dependent phosphorylation. To answer this question, we first created putative CDK phosphorylation site mutants. Rev1 has 7 S/TP sites, which are CDK consensus phosphorylation sites. One of these sites, T740, is in close proximity to a lysine-rich region, which is important for SCF-dependent proteolysis. We created two mutants: T740A and S/TPs to APs, where all S/TPs were replaced with AP. However, both of these mutations did not alter the protein level of Rev1 or confer any cisplatin sensitivities. We also created a mutant where RXXL, the Cdc13-like destruction box [68], was replaced with AXXA. This mutant also did not show an altered protein level (data not shown). These results suggest that CDK may not trigger the destruction of Rev1, in contrast to the findings for Cdc18. Of course, these preliminary studies cannot rule out the possible involvement of CDK in Rev1 destruction, and we plan to explore this aspect further in future studies.

The temporal increase in Rev1 protein levels during G_1_ phase can be attributed to the requirement for Rev1 during the assembly of TLS polymerases. Several recent studies have shown that Rev1 can serve as a polη- or polκ-assembly factor for polζ [32, 53, 69]. We also found that the *rev1* deletion mutation prevented the association of Rev7 with Eso1. Because the amount of Eso1 is much greater than that of Rev1, it is obvious why Rev1 must be highly upregulated during G_1_ phase. However, it is not clear why Rev1 would need to be destroyed at the G_1_/S transition, despite its requirement in TLS. Cdc18 must be destroyed at G_1_/S; otherwise, re-replication from a single origin may occur, and as a result, DNA replication may not occur correctly [64, 70]. In the present study, the Rev1dK mutant, in which the Rev1 protein remains stably expressed during S phase, conferred sensitivity to cisplatin to the cells but did not disrupt any functional domains. Additional mutations in the BRCT motif, the catalytic domain, or the UBM domain increased the cisplatin sensitivity of the Rev1dK mutant. Moreover, Rev7 and Cdc1 successfully interacted with Rev1dK in the immunoprecipitation assay. Thus, we hypothesize that excessive Rev1 protein expression can interfere with TLS. This hypothesis is also supported by the observation that overexpression of wild-type *rev1* from an ectopic promoter conferred sensitivity to cisplatin. Similar inhibition was observed in budding yeast when the Rev1 C-terminal domain was overexpressed [56].

To examine the expression of TLS polymerases in the cell cycle, we employed *cdc25*-synchronized culture. In this synchronized culture experiment, it is difficult to distinguish the cell cycle phase precisely; for example, it can be difficult to distinguish phases before or after the initiation of DNA replication. Therefore, we investigated Rev1 protein levels further in cell cycle mutant-arrested cultures. Rev1 protein levels were highest during G_1_ phase and decreased sharply at the G_1_/S boundary. However, this method also has some limitations. Additionally, we observed an increase in Rev1 protein levels in *cdc21* and *cdc17* mutants, even though these mutants arrest the cell cycle during S/G_2_ phase. We assumed that this unexpected upregulation of Rev1 was caused by DNA damage. Indeed, Rev1 was upregulated upon DNA damage in a Rad3-dependent manner. The cdc17 mutant is known to leave nicked regions in chromosomal DNA under restrictive conditions [71]. These damaged regions provoke the activation of the DNA damage checkpoint. However, induction of DNA damage was not clearly observed in the *cdc21* mutant. In addition, Rad3 is known to be activated in the *cdc20* mutant [72]. Therefore, the unexpected upregulation of Rev1 in *cdc21* may be a result of processes other than DNA damage. It is still necessary to elucidate the mechanisms through which Rev1 protein levels are regulated in *cdc20* and *cdc21* mutants.

**Fig 8:**
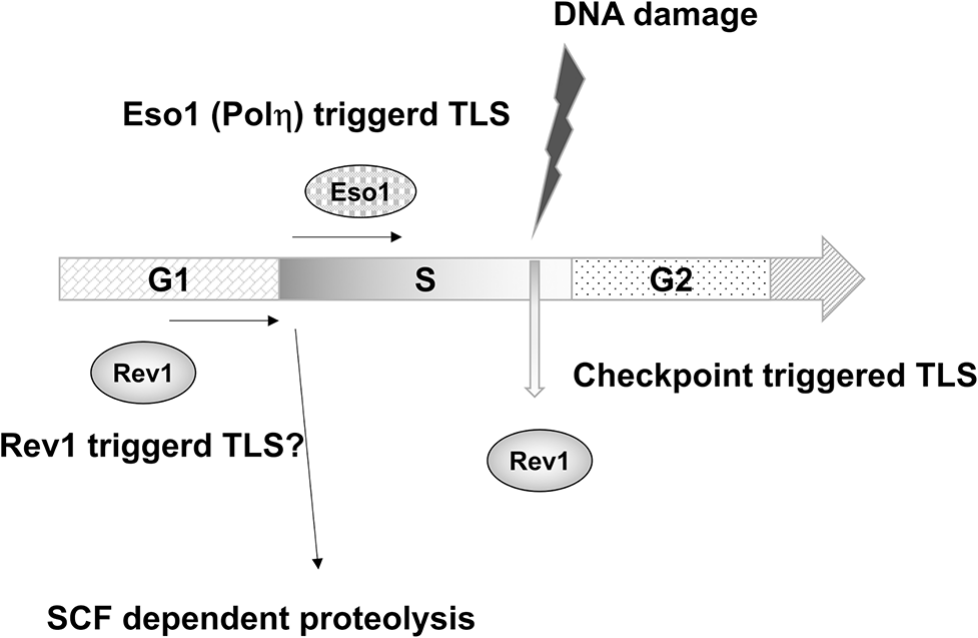
A model for the protein level regulation of Rev1 and TLS. In G1 phase, Rev1 is abundant and Rev1-dependent loading of TLS polymerase may occur. At the onset of S phase, Rev1 is destroyed in a SCF-dependent manner and chromatin-loaded Eso1/polη serves as an initiator of TLS. When DNA is damaged, the DNA structure checkpoint increases the protein level of Rev1 and facilitates polymerase switching among TLS polymerases.

Taking all of the results into consideration, we have proposed a model for Rev1 regulation, as shown in Fig 8. When cells are in the G_1_ phase, Rev1 is abundant compared to Eso1/polη or polζ. In S phase, Eso1/polη becomes abundant and TLS may be conducted mainly by Eso1/polη as reported previously [73]. When the cell experiences DNA damage, Rev1 is upregulated in a Rad3-dependent manner. This upregulation facilitates the complex formation and the switching of TLS polymerase depending on the type of DNA damage. Further analyses are necessary; however, this hypothesis explains the current findings.

## Supporting Information

S1 Fig Eso1 and Kpa1 were abundantly expressed in the chromatin fraction in S phase. (TIF)

S2 Fig Rev1 associated with Rev7. (TIF)

S3 Fig Pop1 associated with Rev1. (TIF)

S4 Fig Construction of *rev1* functional domain mutants. (TIF)

S5 Fig Rev1 association with Cdc1. (TIF)

S6 Fig Protein level of overexpressed Rev1. (TIF)

S7 Fig DNA damage sensitivities of *rev1Δ* and *rad3Δ*. (TIF)

S1 Table Fission yeast strains used in this study. (DOCX)
