# Supplementary figures and images for: The Protein Level of Rev1, a TLS Polymerase in Fission Yeast, Is Strictly Regulated during the Cell Cycle and after DNA Damage
Source: PLoS One. 2015 Jul 6;10(7):e0130000. doi: 10.1371/journal.pone.0130000 (PMC4493104; doi:10.1371/journal.pone.0130000)

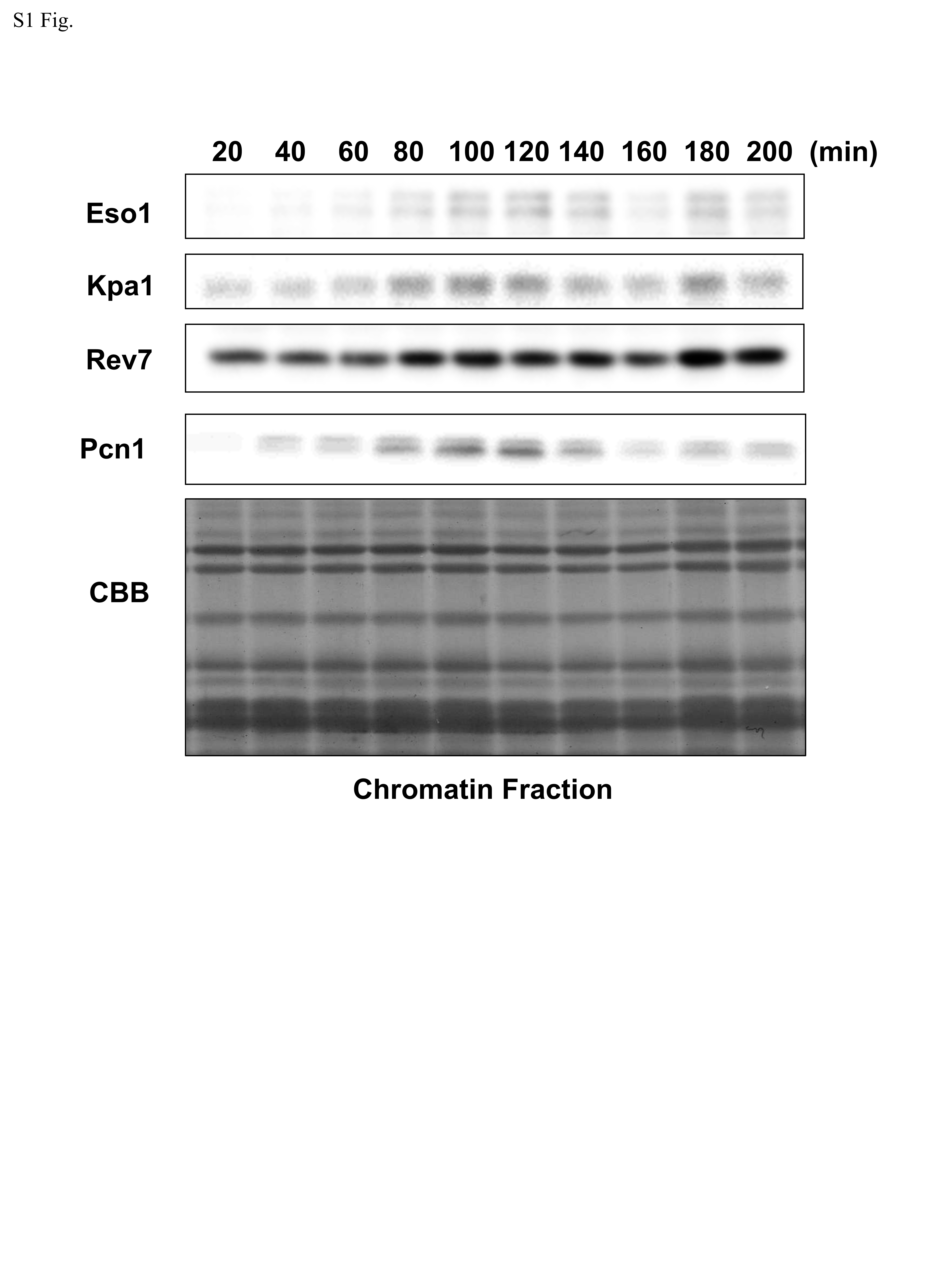

Supplement: S1 Fig — (TIF) [file pone.0130000.s001.TIF]

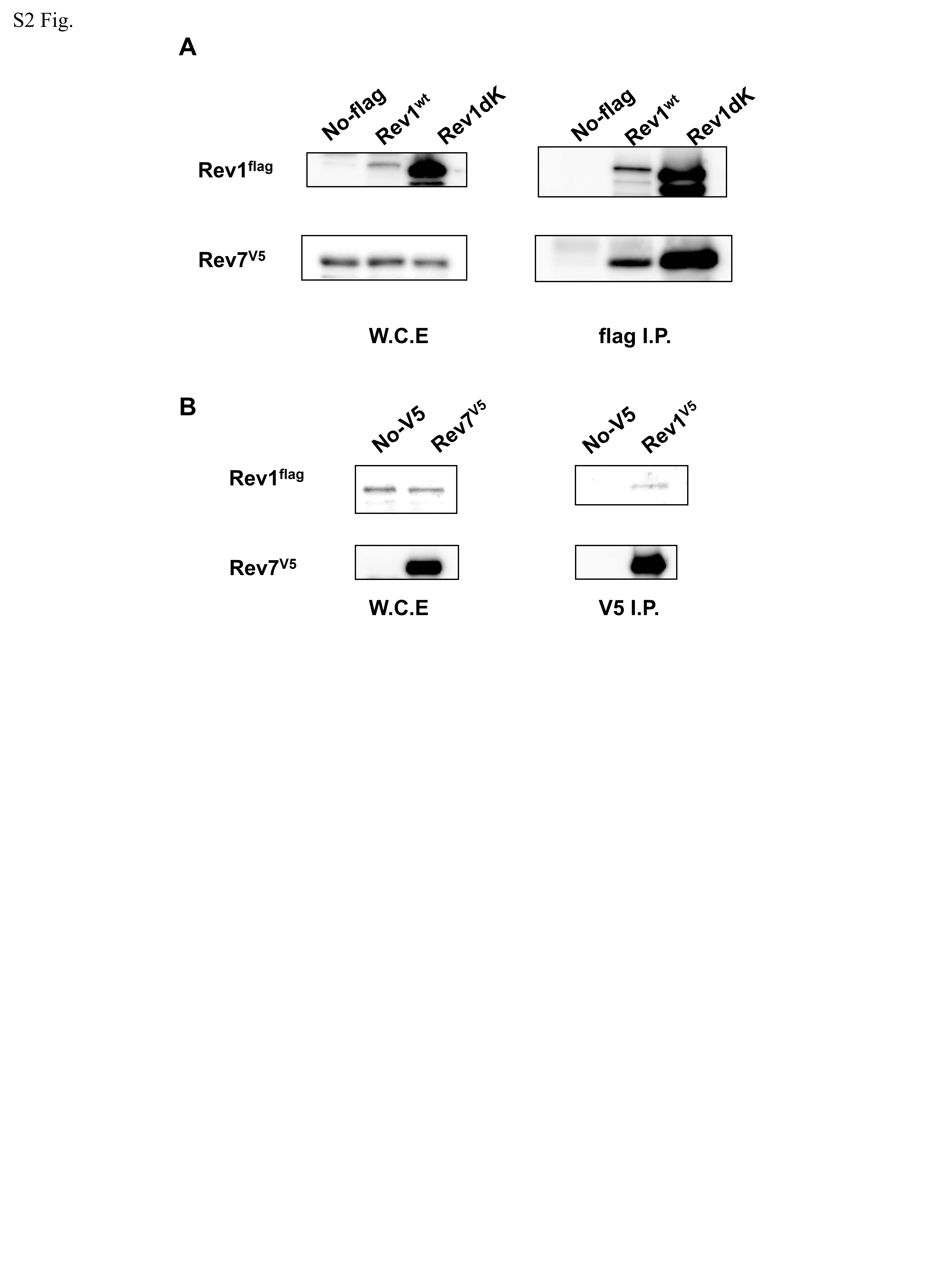

Supplement: S2 Fig — (TIF) [file pone.0130000.s002.TIF]

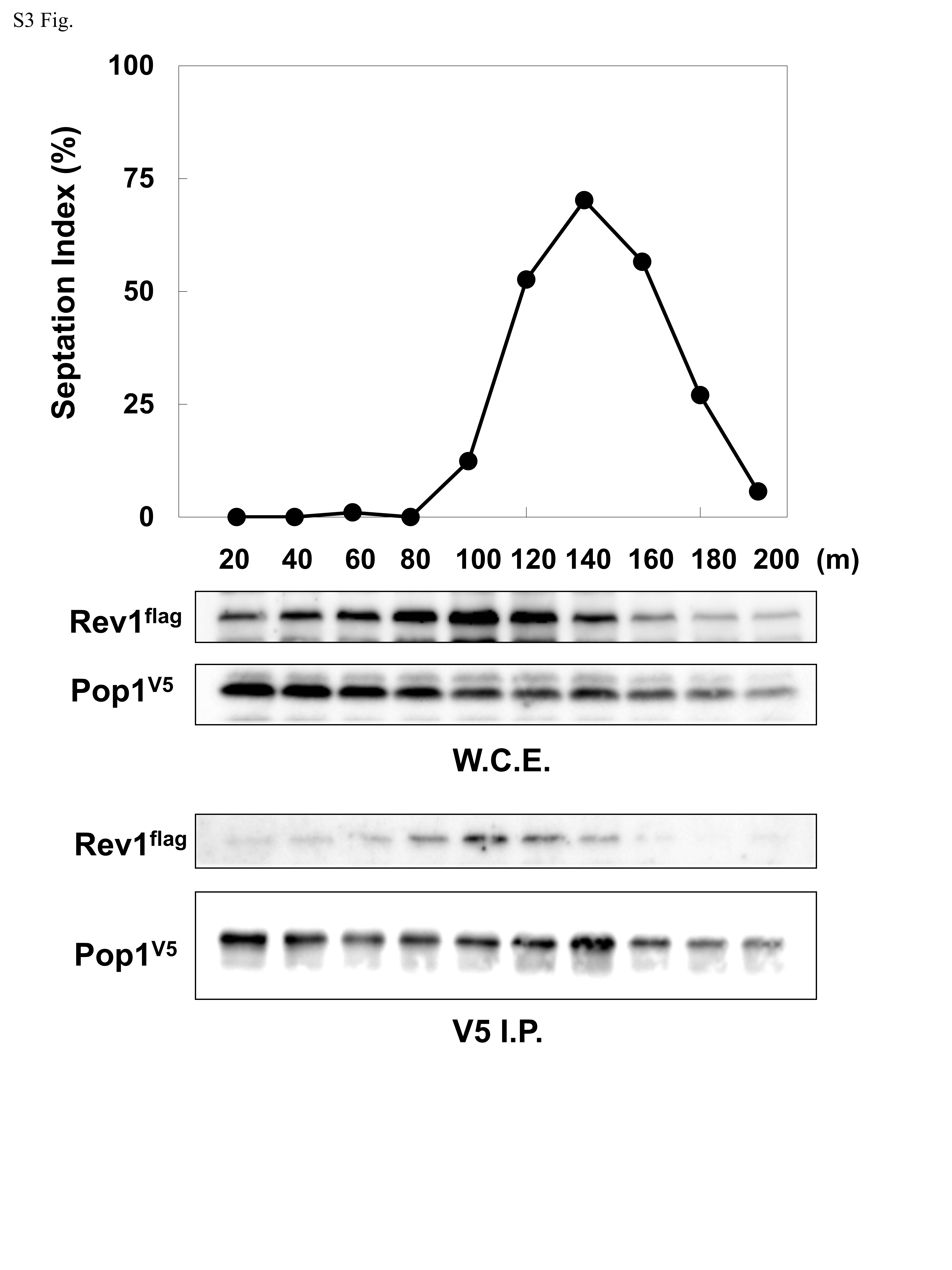

Supplement: S3 Fig — (TIF) [file pone.0130000.s003.TIF]

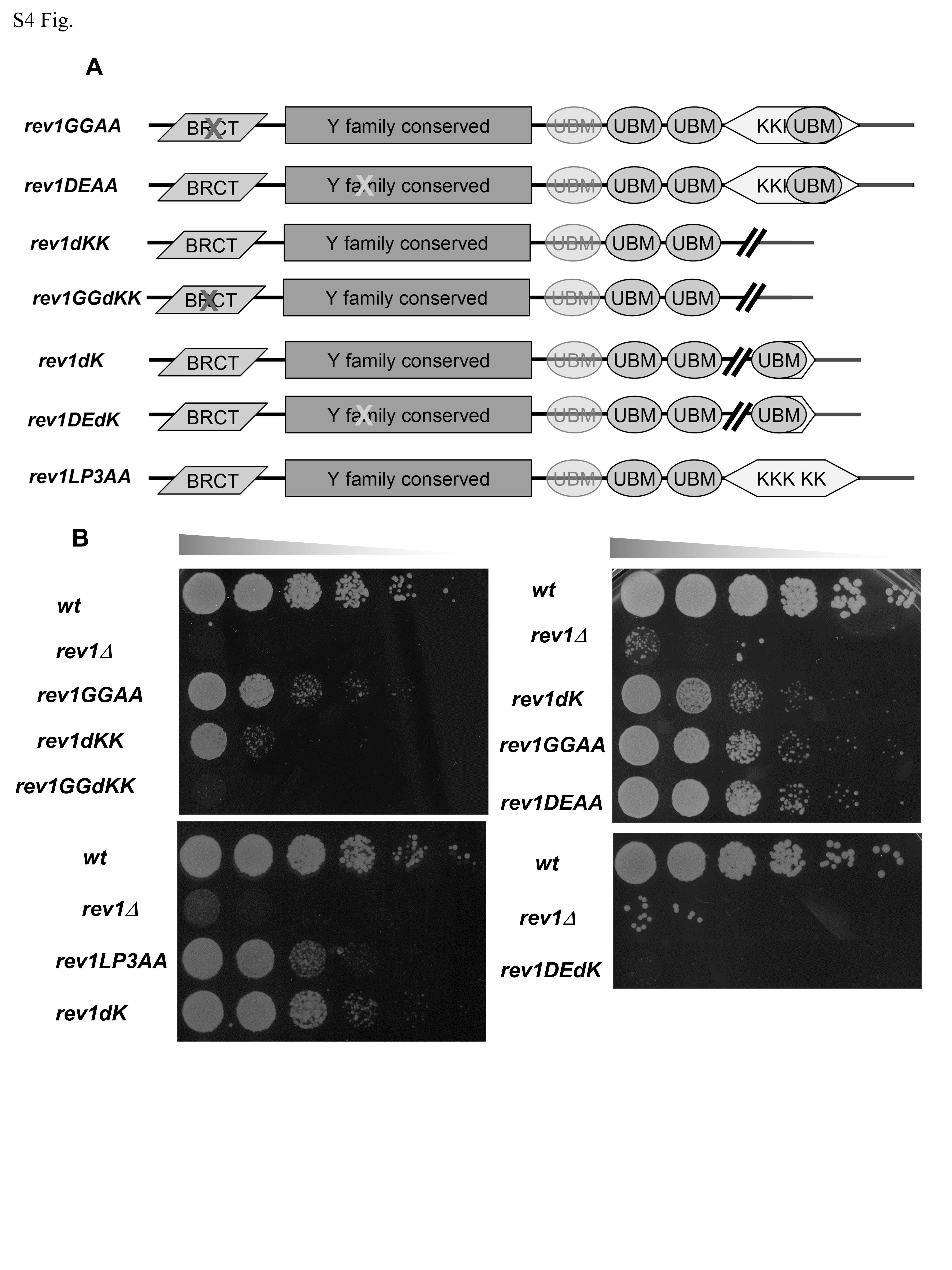

Supplement: S4 Fig — (TIF) [file pone.0130000.s004.TIF]

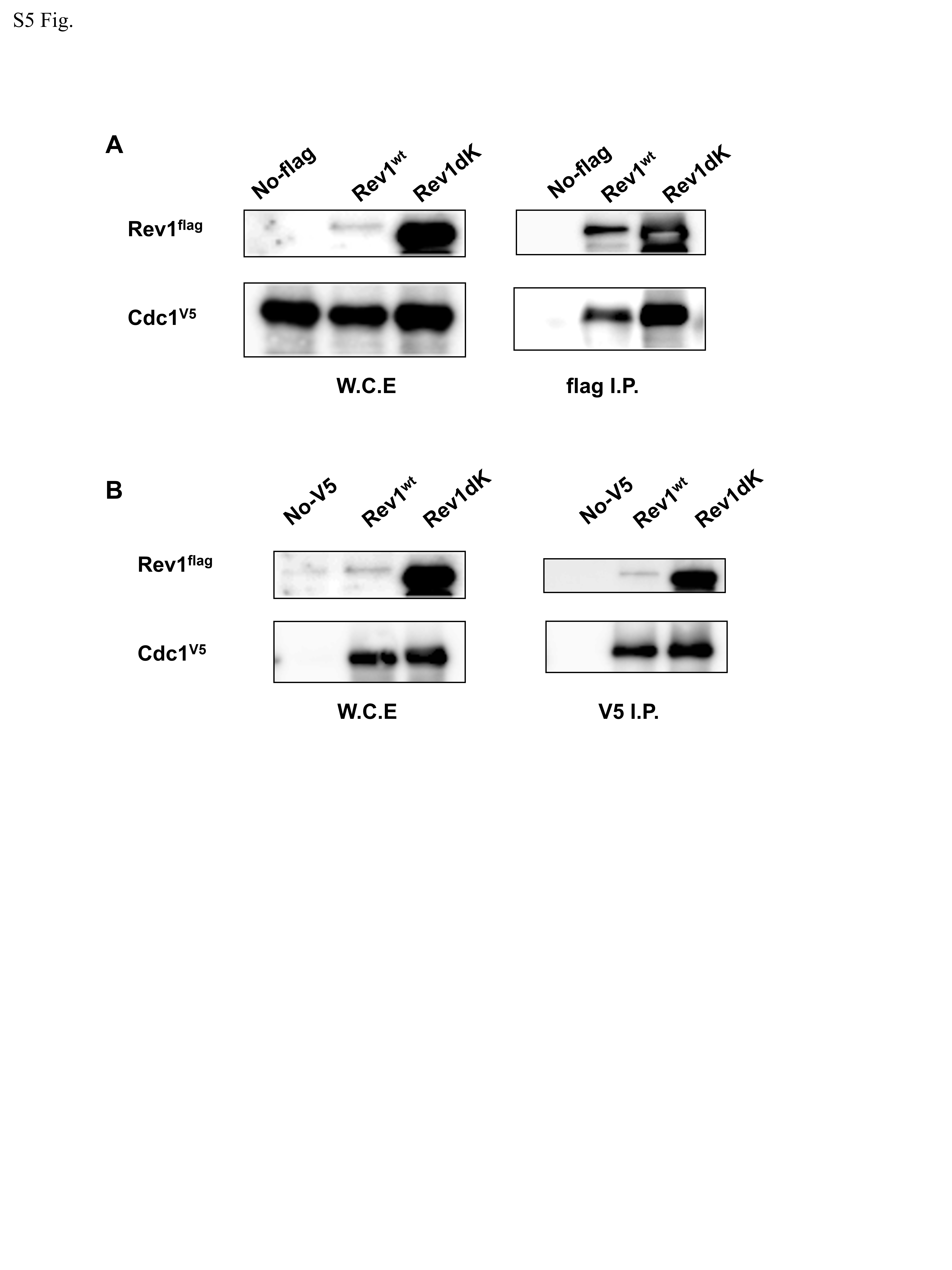

Supplement: S5 Fig — (TIF) [file pone.0130000.s005.TIF]

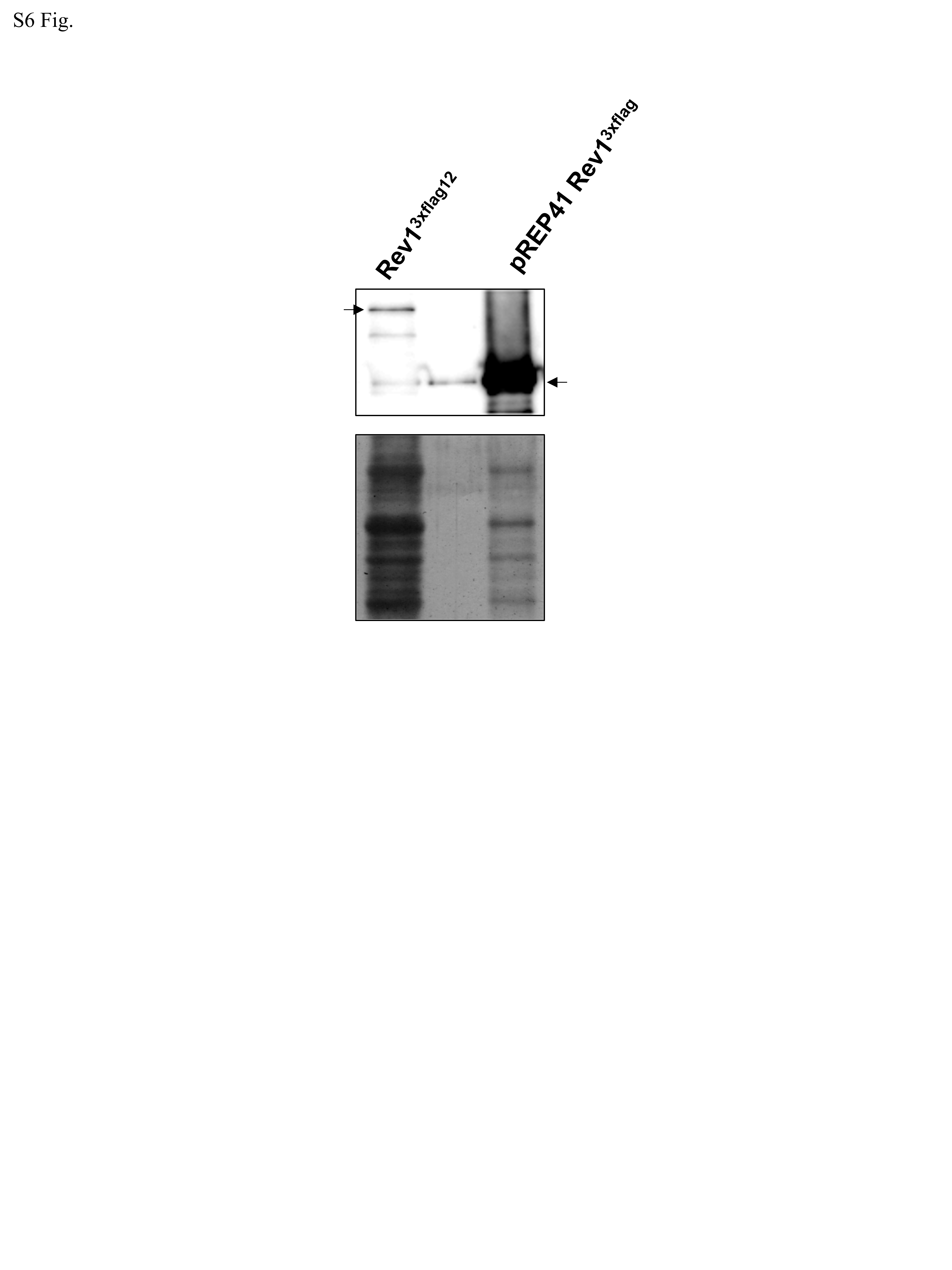

Supplement: S6 Fig — (TIF) [file pone.0130000.s006.TIF]

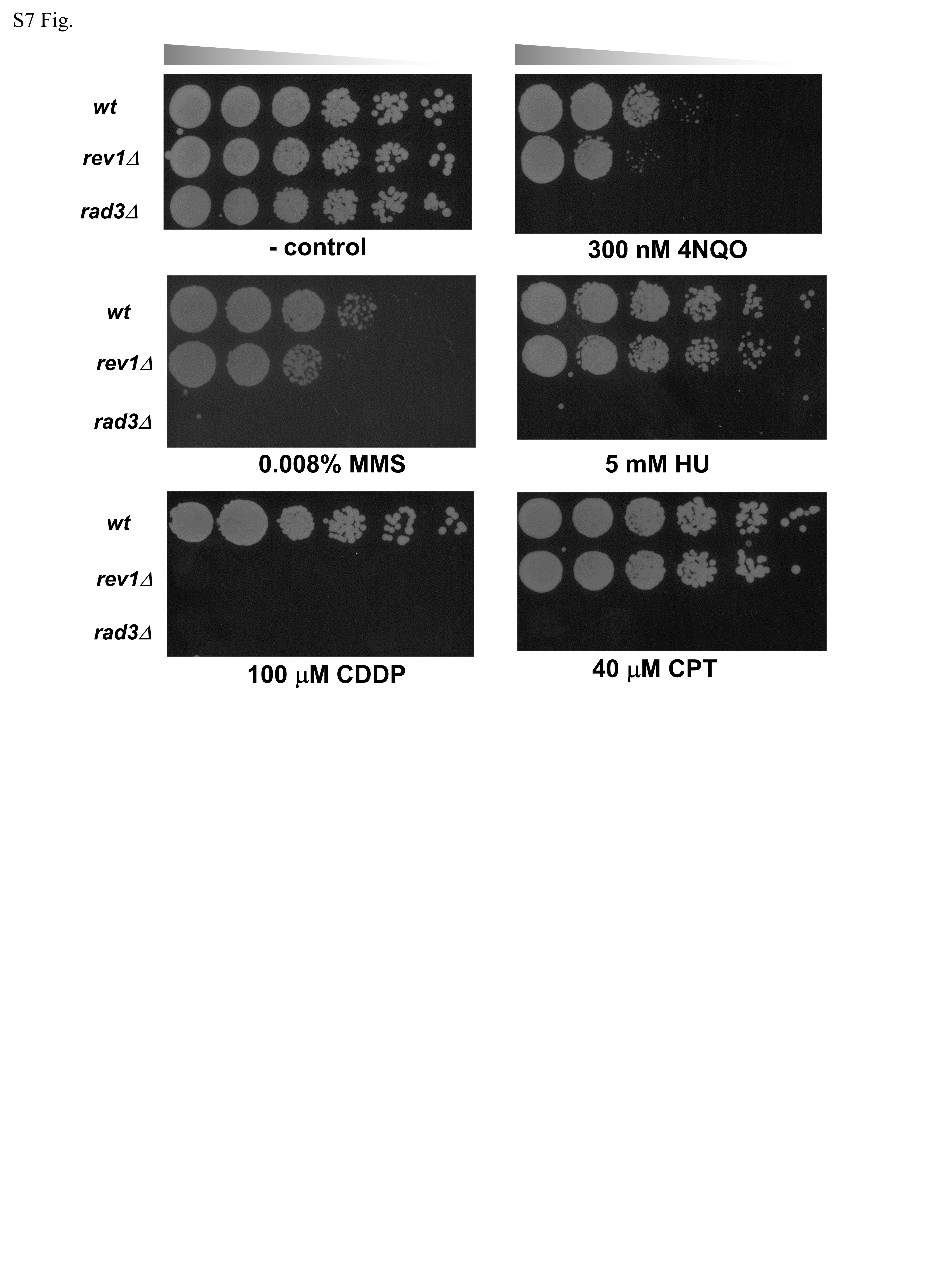

Supplement: S7 Fig — (TIF) [file pone.0130000.s007.TIF]
