# Supplementary material for: The Protein Level of Rev1, a TLS Polymerase in Fission Yeast, Is Strictly Regulated during the Cell Cycle and after DNA Damage
Source: PLoS One. 2015 Jul 6;10(7):e0130000. doi: 10.1371/journal.pone.0130000 (PMC4493104; doi:10.1371/journal.pone.0130000)
Supplement: S1 Table — (DOCX) [file pone.0130000.s008.docx]

**S1 Table. Fission yeast strains used in this study.**

| Strain | Genotype | Source |
| --- | --- | --- |
| 972 | *h^-^* |  |
| *wt h^-^* | *h^-^ ade6-M210 leu1-32 ura4-D18 his3-D1* |  |
| *wt h^+^* | *h^+^ ade6-M216 leu1-32 ura4-D18 his3-D1* |  |
| *rev1Δ* | *h^-^ rev1::his3 leu1-32 ade6-M210 ura4-D18 his3-D1* | This study |
| *rev1dK* | *h^-^ rev1dK leu1-32 ade6-M210 ura4-D18 his3-D1* | This study |
| *rev1dKK* | *h^-^ rev1dKK leu1-32 ade6-M210 ura4-D18 his3-D1* | This study |
| *cam1-rev1^flag^* | *h^-^ rev1::kanR::cam1-rev1flag:ura4 leu1-32 ade6-M210 ura4-D18 his3-D1* | This study |
| *rad3Δ* | *ｈ^+^ rad3::ura4 leu1-32 ade6-M210 ura4-D18* |  |
| *eso1^Δpolh^* | *h^-^ eso1::ctf7 leu1-32 ade6-M210 ura4-D18 his3-D1* | [[28](#_ENREF_28)] |
| *eso1^myc^* | *h^-^ eso1::eso1-myc13:his3 leu1-32 ade6-M210 ura4-D18 his3-D1* | This study |
| *eso1^V5^* | *h^-^ eso1::eso1-V5x8:ura4 leu1-32 ade6-M210 ura4-D18 his3-D1* | This study |
| *rev1^flag^* | *h^-^ rev1::rev1-3xflag12:his3 leu1-32 ade6-M210 ura4-D18 his3-D1* | This study |
| *rev7^flag^* | *h^-^ rev7::rev7-3xflag:ura4 leu1-32 ade6-M210 ura4-D18 his3-D1* | This study |
| *rev7^V5^* | *h^-^ rev7::rev7-V5x8:ura4 leu1-32 ade6-M210 ura4-D18 his3-D1* | This study |
| *rev1dK^flag^* | *h^-^ rev1::rev1dK-3xflagx12:his3 leu1-32 ade6-M210 ura4-D18 his3-D1* | This study |
| *rev1dKK^flag^* | *h^-^ rev1::rev1dKK-3xflagx12:his3 leu1-32 ade6-M210 ura4-D18 his3-D1* | This study |
| *kpa1^flag^* | *h^-^ kpa1::kpa1-3xflag:his3 leu1-32 ade6-M210 ura4-D18 his3-D1* | This study |
| *pop1Δ* | *h^-^ pop1::ura4 ade6-M210 leu1-32 ura4-D18 his3-D1* | This study |
| *pop2Δ* | *h^-^ pop2::ura4 ade6-M210 leu1-32 ura4-D18 his3-D1* | This study |
| *pop1^V5^* | *h^-^ pop1::pop1-V5x16:ura4 ade6-M210 leu1-32 ura4-D18 his3-D1* | This study |
| *pop2^V5^* | *h^-^ pop2::pop2-V5x16:ura4 ade6-M210 leu1-32 ura4-D18 his3-D1* | This study |
| *cdc10* | *h^−^ cdc10-M17 ura4-D18 leu1-32 ade6-M216* |  |
| *cdc17* | *h^−^ cdc10-K42 ura4-D18 leu1-32 ade6-M216* |  |
| *cdc20* | *h^−^ cdc20-M10 ura4-D18 leu1-32 ade6-M216* |  |
| *cdc21* | *h^−^ cdc21-M68 ura4-D18 leu1-32 ade6-M216* |  |
| *cdc22* | *h^+^ cdc22-M45 ura4-D18 leu1-32 ade6-M210* |  |
| *cdc25* | *h^+^ cdc25-22 leu1-32 ade6-M216* |  |
| *mts2-U31* | *h^-^ mts2::mts2-U31:ura4 ade6-M210 leu1-32 ura4-D18 his3-D1* | This study |
| *mts3-U32* | *h^-^ mts3::mts3-U32:ura4 ade6-M210 leu1-32 ura4-D18 his3-D1* | This study |

Strains for which the sources were not specified are lab stock and publically available (e.g., those from NBRP).
